# Developmental and hemocytological effects of ingesting Fukushima’s radiocesium on the cabbage white butterfly ***Pieris rapae***

**DOI:** 10.1038/s41598-018-37325-9

**Published:** 2019-02-22

**Authors:** Wataru Taira, Mariko Toki, Keisuke Kakinohana, Ko Sakauchi, Joji M. Otaki

**Affiliations:** 1 The BCPH Unit of Molecular Physiology, Department of Chemistry, Biology and Marine Science, Faculty of Science, University of the Ryukyus, Okinawa, 903-0213 Japan; 2 Center for Research Advancement and Collaboration, University of the Ryukyus, Okinawa, 903-0213 Japan

## Abstract

High morphological abnormality and mortality rates have been reported in the pale grass blue butterfly, *Zizeeria maha*, since the Fukushima nuclear accident. However, it remains uncertain if these effects are restricted to this butterfly. Here, we evaluated the effects of ingesting cabbage leaves grown with contaminated soils from Fukushima on the development and hemocytes of the cabbage white butterfly, *Pieris rapae*. Contaminated cabbage leaves containing various low levels of anthropogenic ^134^Cs and ^137^Cs radioactivity (less than natural ^40^K radioactivity) were fed to larvae from Okinawa, the least contaminated locality in Japan. Negative developmental and morphological effects were detected in the experimental groups. The cesium (but not potassium) radioactivity concentration was negatively correlated with the granulocyte percentage in hemolymph, and the granulocyte percentage was positively correlated with the pupal eclosion rate, the adult achievement rate, and the total normality rate. These results demonstrated that ingesting low-level radiocesium contaminants in Fukushima (but not natural radiopotassium) imposed biologically negative effects on the cabbage white butterfly, as in the pale grass blue butterfly, at both cellular and organismal levels.

## Introduction

Information on the biological impacts of ionizing radiation has been gathered for nearly a century since the discovery of X-ray-induced mutations by Hermann J. Muller in the fruit fly^1,2^ and by Lewis J. Stadler in barley and maize^3,4^. Adverse biological effects of naturally high background radiation have been documented^5^, but presently, radiation-based technologies are widespread in society, which leads to the relatively high possibility of radiation exposure, whether intentional or accidental, for anyone. An “intentional” radiation exposure that one may encounter is medical X-ray exposure for computed tomography (CT). Although it has been understood that medical exposure is necessary to obtain critical information on patients’ pathogenesis and that the potential adverse effects from the exposure are negligible under well-controlled procedures, recent findings that even a single CT scan causes chromosomal damage^6^ and that cumulative exposures increase cancer risks significantly^7–9^ suggest that current knowledge on biological responses to radiation exposure is far from complete.

Major “accidental” (unintentional) exposure comes from environmental radioactive pollution by nuclear bomb tests and failures of nuclear power plants. The Chernobyl nuclear accident in April 1986 and the Fukushima nuclear accident in March 2011 have been recognized as the two worst nuclear accidents in the history of humankind^10^. Biological impacts of the Chernobyl accident have been documented in birds^11–16^, and more recently, those data were examined in a meta-analysis^17^. Several field studies have documented the effects of the Fukushima nuclear accident on various organisms: bird and arthropod populations^18–20^, gall-forming aphids^21^, Japanese monkeys^22–25^, barn swallows^26^, goshawks^27^, rice plants^28,29^, fir trees^30^, red pine trees^31^, and populations of intertidal species including the rock shell^32^. At the biochemical level, stress responses might have been induced in cattle in the contaminated areas^33^. Fukumoto and colleagues have recently reported changes in gene expression in the small intestine of pigs^34^, DNA damage in bovine lymphocytes^35^, and enhanced spermatogenesis in large Japanese field mice^36^. Another independent group has also reported chromosomal aberrations in large Japanese field mice^37^. In contrast, some studies have reported no detectable effects. For example, simulation experiments using ^137^Cs irradiation in mice did not show any detectable change in litter size, sex ratio, and biokinetics^38^. Mammalian testes from bull, boar, inobuta (wild boar and domestic pig hybrid), and large Japanese field mice in the contaminated areas did not show any noticeable abnormalities^39–41^.

We have been using the pale grass blue butterfly *Zizeeria maha* as an experimental system^42,43^ to examine the biological effects of the Fukushima nuclear accident^44–58^. In one of the most important experiments, larvae from Okinawa, the least contaminated locality in Japan, were fed the contaminated wild-harvested leaves of *Oxalis corniculata* from Fukushima, resulting in dose-dependent abnormality and mortality^44,46,47,51^. This dose-response relationship successfully fitted a sigmoidal Weibull function or power function curve^51^. Use of the contaminated wild leaves from Fukushima and the non-contaminated larvae from Okinawa is an important experimental strategy to understand what has happened in Fukushima, without emphasizing the identification of causal substances and mechanisms. Because small but detectable changes in abnormality and mortality rates were demonstrated at low-dose levels in the pale grass blue butterfly, one of the important questions to resolve is whether this high sensitivity to low-level contamination is restricted to this butterfly species or not. To the best of our knowledge, there have been no such feeding experiments performed in other species in Fukushima research. Additionally, hemocytological examinations at low doses, which are often said to occur in response to radiation exposures in mammals^22–24,26,33^, are entirely absent in the case of the pale grass blue butterfly in Fukushima. Hemocytological examinations may be able to pinpoint environmental stress effects at the cellular level even when there are few morphological abnormalities at the organismal level.

Here, we used the cabbage white butterfly, *Pieris rapae*, to investigate the effects of ingestion of cabbage leaves that were grown with contaminated Fukushima soils. Our choice of this butterfly is three-fold. First, it is a different species from the pale grass blue butterfly but may be similar enough to reproduce the previous experiments as a lepidopteran species, as a first step to examine generality of the previous experiments. Second, the cabbage white butterfly is more suitable for hemocytological examinations than the pale grass blue butterfly because it is possible to obtain much hemolymph with easier manipulations due to its larger body size. Importantly, hemocytotological data for this butterfly are available; in the last instar larvae, and highly likely in prepupae, of the cabbage white butterfly, there are only three kinds of hemocytes in the hemolymph, granulocytes, plasmatocytes, and prohemocytes^59^, providing us with a simple system to study. Oenocytoids and spherulocytes, which are often found in other lepidopteran hemolymph, are not found in the cabbage white butterfly^59^. Third, the host plant leaves of the cabbage white butterfly (i.e., cabbage) are easy to cultivate because cabbage is a well-established agricultural plant.

**Figure 1:**
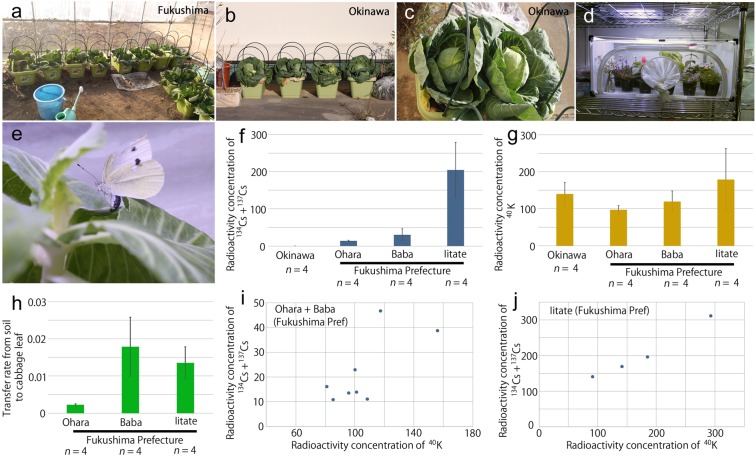
Cabbage cultivation and radioactivity concentrations of cesium and potassium in cabbage. (**a**–**c**) Cabbage cultivation in Fukushima and Okinawa. (**d**) Entire setup for egg collection from field-caught females in the laboratory. (**e**) Oviposition in the container in the laboratory. (**f**) Radioactivity concentrations of ^134^Cs and ^137^Cs [Bq/kg] in the soil used to cultivate cabbage. (**g**) Radioactivity concentrations of ^40^K [Bq/kg] in the soil used to cultivate cabbage. (**h**) Transfer rate of cesium from soil to cabbage leaves (radioactivity of cabbage leaves divided by radioactivity of soil). (**i**) Scatter plot of radioactive concentrations of cesium and potassium [Bq/kg] in cabbage leaves from Ohara and Baba, Fukushima Prefecture. (**j**) Scatter plot of radioactive concentrations of cesium and potassium [Bq/kg] in cabbage leaves from Iitate, Fukushima Prefecture. Note the difference in the cesium levels (the vertical axis) from the previous plot (**i**).

Accordingly, we cultivated cabbage using contaminated Fukushima soils (Fig. 1a–c). The contaminated leaves were fed to larvae of the cabbage white butterfly, which were obtained from females that were caught in Okinawa, the least contaminated locality in Japan (Fig. 1d,e). We examined the following 5 developmental factors: pupal eclosion rate, adult achievement rate, total normality rate, larval period, and pupal period (see Methods for definitions). Adult abnormality rate and adult abnormality score were also used when appropriate. Additionally, adult male and female forewing sizes were examined. Importantly, we also examined 3 hemocytological factors, including the percentages of granulocytes, plasmatocytes, and prohemocytes in hemolymph. Granulocytes and plasmatocytes are functional cells in the insect immune system, and prohemocytes are immature cells that differentiate into plasmatocytes and then to granulocytes^59–61^. Possible correlations of these factors with radioactivity concentrations of anthropogenic radiocesium (^134^Cs + ^137^Cs), naturally occurring radiopotassium (^40^K), and their summation (^134^Cs + ^137^Cs + ^40^K) in cabbage leaves were examined.

## Results

### Radioactivity concentrations in cultivated cabbage leaves

Soil samples were obtained from 4 different localities in Okinawa and Fukushima prefectures with various levels of radioactive contamination: Okinawa (control), Ohara (relatively low levels of soil contamination), Baba (relatively intermediate levels), and Iitate (relatively high levels). Using these soils, we successfully obtained 4 groups of cabbage leaves containing various radioactivity concentrations of ^134^Cs, ^137^Cs, and ^40^K (Table S1). Among them, cabbage leaves from the Iitate soil sample contained relatively high levels of radiocesium (Fig. 1f). In contrast, radioactivity concentrations of ^40^K in cabbage leaves did not vary much among the 4 locality groups (Fig. 1g), but the radiopotassium levels were higher than the radiocesium levels with the exception of the Iitate group. Partly because ^40^K occurs naturally, the levels of radioactive cesium contamination in these samples that were comparable to those of ^40^K may be considered very low or even negligible in current public policy^62^. However, we stress that the contamination effects on organisms must be determined experimentally.

Transfer rates of radioactivity from soil to cabbage leaf (wet) varied among the 3 localities (Fig. 1h). This variability likely stemmed from various soil compositions, which were not controlled for in this study. Thus, subsequent statistical analyses were performed based on radioactivity concentrations of cabbage leaves but not of soils.

A linear relationship was detected between the radioactivity concentrations of radiocesium (^134^Cs + ^137^Cs) and radiopotassium (^40^K) in cabbage leaves; therefore, the Ohara and Baba groups were examined together (*n* = 8, Pearson correlation coefficient *r* = 0.71, *P* = 0.047) (Fig. 1i), whereas the Iitate group was examined independently (*n* = 4, *r = *0.99, *P* = 0.0098) (Fig. 1j). This approach is because the data points were distributed at different levels, suggesting that these two groups might have behaved differently. Alternatively, when all data points were examined together, a reasonably high linear relationship was also obtained (*n* = 12, *r = *0.85, *P* = 0.0004).

### Quality check for the rearing system

**Figure 2:**
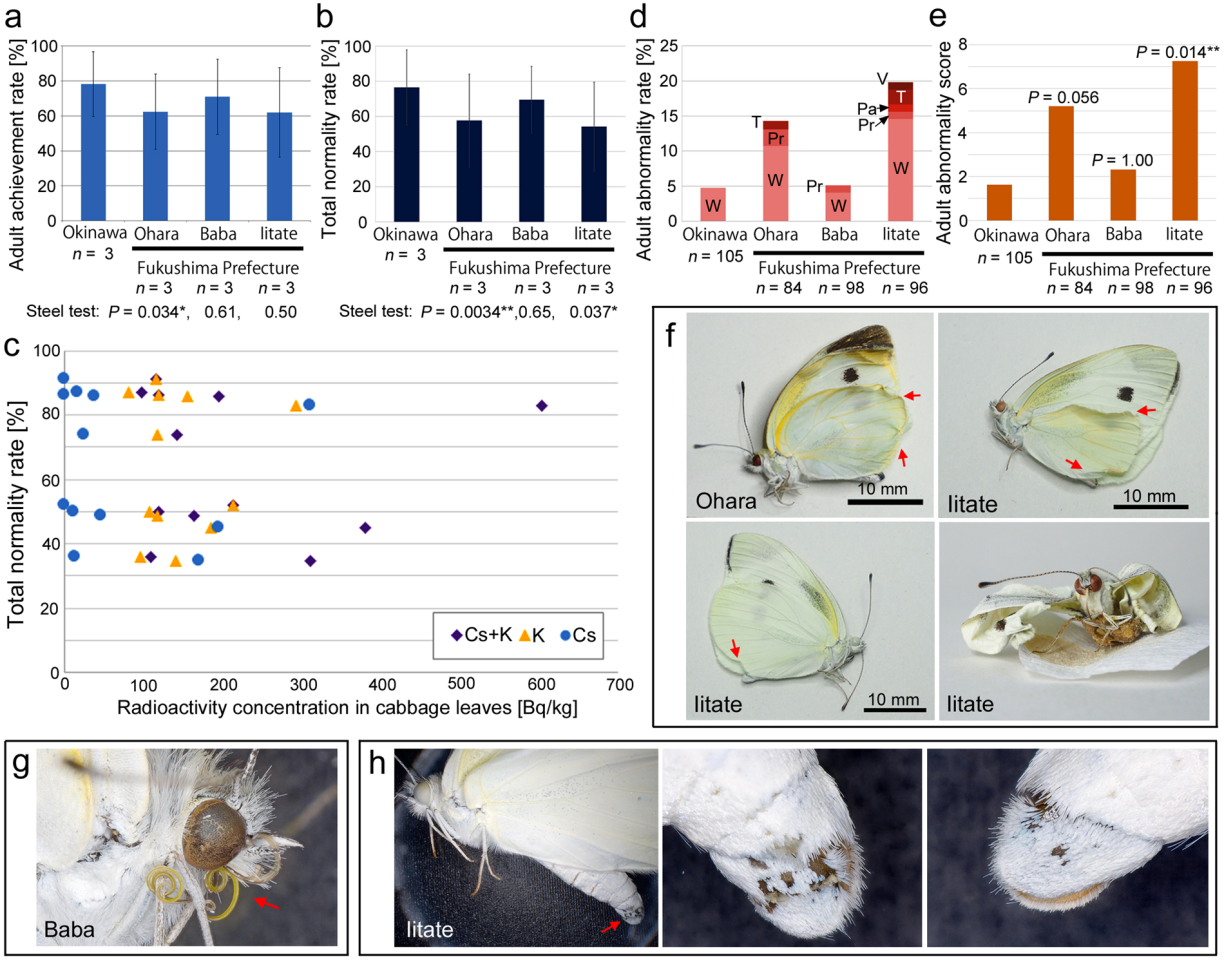
Morphological abnormalities and related factors in the cabbage white butterflies that consumed contaminated leaves. (**a**,**b**) Adult achievement rate (%) (**a**) and the total normality rate (%) (**b**). Mean values of 3 trials are shown with standard deviation bars. *P*-values of Steel tests (in reference to the Okinawa group) are also shown. (**c**) Scatter plots of the total normality rate (%) against the radioactivity concentration in cabbage leaves [Bq/kg]. Each dot represents a single trial. Different shapes and colors of dots indicate different species of radionuclides in obtaining radioactivity concentrations in cabbage leaves. (**d**) Adult abnormality rate (%) and its abnormality profile. Mean values are shown. Proportions of abnormalities in wings (W), proboscises (Pr), trunk (T), palpi (Pa), and valva (V) are indicated. (**e**) Adult abnormality score based on the scoring system. *P*-values were obtained from Steel tests in reference to the Okinawa group. (**f**) Examples of wing abnormalities from the Ohara and Iitate groups. (**g**) Examples of proboscis abnormality from the Baba group. (**h**) A case of abnormality in left valva from the Iitate group.

To assess the quality of our rearing system, the adult achievement rate (proportion of individuals that became adults among the reared larvae) was obtained (Fig. 2a; Table S2). The Okinawa control group showed an adult achievement rate of 78.2 ± 18.4 [%] (mean ± SD), demonstrating that almost 80% of the reared individuals successfully became adults. This result was considered reasonably high quality as a rearing control. The Ohara, Baba, and Iitate groups showed smaller rates, and the Ohara group showed a statistically significant difference in the adult achievement rate from the Okinawa group (*P* = 0.034; Steel test). Similarly, the total normality rate (proportion of normal adult individuals among the reared larvae) was obtained (Fig. 2b; Table S2). The Okinawa group showed a total normality rate of 76.5 ± 21.4 [%] (mean ± SD), demonstrating again a reasonable value for evaluating any influences in the other groups. Similar to the adult achievement rate, the Ohara, Baba, and Iitate groups exhibited smaller total normality rates, and the Ohara and Iitate groups showed statistically significant differences in the total normality rate from the Okinawa group (*P* = 0.0034 and 0.037, respectively; Steel test).

Because radiocesium values of cabbage leaves varied even among the cabbage samples from the same locality, the total normality rate of all samples was plotted against the radioactivity concentration of cesium, potassium, or their summation in cabbage leaves (Fig. 2c). No clear relationship could be deciphered from the scatter plots, but again, the sample distribution in response to radiocesium of roughly more than 100 Bq/kg appeared to be different from that of the lower levels. Together with the K-Cs plots above (Fig. 1i,j), the Iitate samples were considered to have behaved differently from the rest of the samples due to different radioactivity levels and were excluded in the subsequent correlation analyses.

### Morphological abnormalities

Morphological abnormalities from individuals that ate the contaminated cabbage leaves included deformations of wings, proboscises (mouth parts), palpi (a pair of sensory appendages), trunk (thorax), and valva (a male sex organ at the abdominal tip) (Fig. 2d–h; Table S3). In addition to the fact that abnormality rates of the Ohara and Iitate groups were higher than that of the Okinawa group, the Ohara and Iitate groups included various types of abnormalities (Fig. 2d). This result contrasts with the control Okinawa group that had only wing abnormalities. When each type of abnormality was given scores that reflected the frequency of that abnormality across all samples, the Ohara group and Iitate groups had abnormality scores 3.3 and 3.6 times higher, respectively, than that of the Okinawa group (Fig. 2e; Table S3). The difference between the Okinawa and Iitate groups was statistically significant (*P* = 0.014; Steel test). Examples of the morphological abnormalities are shown in Fig. 2f–h.

### Developmental factors

In addition to the adult achievement rate and the total normality rate discussed above, we obtained 3 additional developmental factors (i.e., the pupal eclosion rate, the larval period, and the pupal period) for the Okinawa, Ohara, Baba, and Iitate groups (Table S2). Furthermore, the male and female forewing sizes were measured (Table S4). These 7 factors were examined for possible relationships with the levels of 3 kinds of radioactivity concentrations (i.e., ^134^Cs + ^137^Cs, ^40^K, and their summation). Thus, in total, 21 pairs were examined simultaneously for their possible relationships.

**Figure 3:**
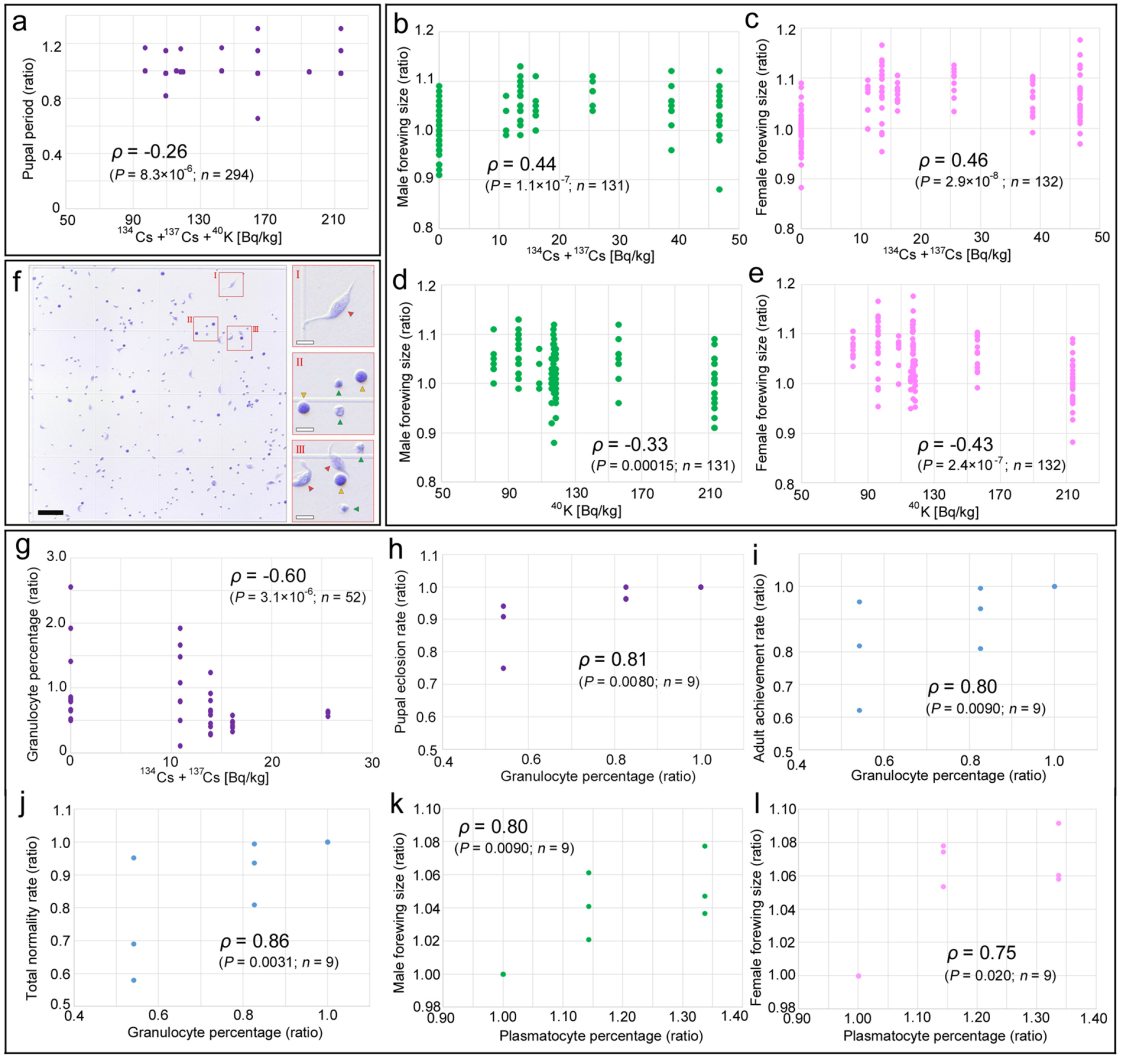
Scatter plots and Spearman correlation coefficients between two factors. There are overlapping points in these plots. (**a**) Pupal period and radioactivity concentrations of cesium and potassium in cabbage leaves. Pupal period was recorded in days, and thus, there were many individuals that had the same values. (**b**) Male forewing size and radioactivity concentration of cesium in cabbage leaves. (**c**) Female forewing size and radioactivity concentration of cesium in cabbage leaves. (**d**) Male forewing size and radioactivity concentration of potassium in cabbage leaves. (**e**) Female forewing size and radioactivity concentration of potassium in cabbage leaves. (**f**) Hemocyte images on the hematological cell counter. Three areas I, II, and III are enlarged at the right side. Red, yellow, and green arrowheads indicate plasmatocytes, granulocytes, and prohemocytes, respectively. The black scale bar indicates 100 μm, and the white scale bars indicate 20 μm. (**g**) Granulocyte percentage and radioactivity concentration of cesium in cabbage leaves. (**h**) Pupal eclosion rate and granulocyte percentage. (**i**) Adult achievement rate and granulocyte percentage. (**j**) Total normality rate and granulocyte percentage. (**k**) Male forewing size and plasmatocyte percentage. (**l**) Female forewing size and plasmatocyte percentage.

Among these, only 5 pairs showed statistically significant *P*-values (Fig. 3). Pupal period was weakly negatively correlated with the summation of cesium and potassium (Spearman correlation coefficient *ρ* = −0.26, *P* = 8.3 × 10^−6^) (Fig. 3a), but its low coefficient and scatter plot were not very sound in terms of a linear relationship. Radiocesium was positively correlated with the male forewing size (*ρ* = 0.44, *P* = 1.1 × 10^−7^) (Fig. 3b) and with the female forewing size (*ρ* = 0.46, *P* = 2.9 × 10^−8^) (Fig. 3c). In contrast, radiopotassium was negatively correlated with the male forewing size (*ρ* = −0.33, *P* = 0.00015) (Fig. 3d) and with the female forewing size (*ρ* = −0.43, *P* = 2.4 × 10^−7^) (Fig. 3e). However, these cases showed low correlation coefficients, and their biological significance is not well understood.

### Hemocyte percentages

After counting the numbers of 3 types of hemocytes (Fig. 3f), their percentages among the whole hemocyte fraction were obtained (Table S5), and their possible correlations were examined with 3 kinds of radioactivity concentrations as above. Among these 9 pairs, a statistically significant correlation was obtained in only one case: the granulocyte percentage and radiocesium were reasonably negatively correlated (*ρ* = −0.60, *P* = 3.1 × 10^−6^) (Fig. 3g), suggesting that radiocesium contributed to negative biological effects.

To understand possible effects of granulocytes or other hemocytes in development, the 3 hemocyte percentages were examined for their possible linear relationships with the 7 developmental factors mentioned above. Among these 21 pairs, statistically significant relationships were obtained in 5 cases. The granulocyte percentage was significantly positively correlated with the pupal eclosion rate (*ρ* = 0.81, *P* = 0.0080) (Fig. 3h), the adult achievement rate (*ρ* = 0.80, *P* = 0.0090) (Fig. 3i), and the total normality rate (*ρ* = 0.86, *P* = 0.0031) (Fig. 3j). The plasmatocyte percentage was significantly positively correlated with the male forewing size (*ρ* = 0.80, *P* = 0.0090) (Fig. 3k) and the female forewing size (*ρ* = 0.75, *P* = 0.020) (Fig. 3l).

## Discussion

Our experimental strategy was to feed contaminated cabbage leaves that were cultivated in Fukushima to the larvae of the cabbage white butterfly from Okinawa. This strategy is similar to the previous internal exposure studies using the pale grass blue butterfly^44–47^. However, the present study differs from the previous studies in the following four important ways: (1) a different species of butterfly was used to test general applicability of the previous results, (2) the host plant leaves were not wild-harvested but rather cultivated to control the quality of plants using the contaminated and non-contaminated soils, (3) hemocytological factors in addition to developmental factors and forewing sizes were examined, and (4) the radioactivity concentrations of ^40^K were measured in addition to those of ^134^Cs and ^137^Cs.

With respect to the first point, this study partly answered the question of the high sensitivity of the pale grass blue butterfly in the previous internal exposure experiments^44–47^. The contamination levels of wild *Oxalis* leaves from Atami (2.5 Bq/kg), Musashino (6.4 Bq/kg), Kashiwa (48 Bq/kg), Koriyama (117 Bq/kg) and Motomiya (161 Bq/kg) used in Nohara *et al*. (2014)^47^ are roughly similar to the contamination levels of the cabbage leaves used in this study. Negative effects in the experimental groups with respect to morphological abnormalities and the granulocyte percentage were detected in the present study. Therefore, we conclude that the cabbage white butterfly is roughly as sensitive to radiation concentration of food as the pale grass blue butterfly. However, these species would differ in genetic variation, population size, and other points, making a precise species comparison difficult.

Although there is no precise record of the population dynamics of the cabbage white butterfly in contaminated areas immediately after the Fukushima nuclear accident, Nagahata (2015)^63^ reported that the population of the cabbage white butterfly appeared to have declined dramatically in highly contaminated areas, although there were no numerical data presented. Nagahata (2015)^63^ speculated that this decline would be partly because residents were evacuated and stopped cultivating cruciferous plants but may also be partly because of the biologically adverse effects of radiation exposures.

Our conclusion suggests the possibility that the high sensitivity may be generalizable to lepidopteran insects and to some other animals. However, it should be noted that the quality of radionuclides that were ingested by the two species of butterflies may be different. Radioactivity of wild-collected plant leaves in the previous studies may be found not only inside the plants but also on the surface of leaves, on which radioactive particles or soil dusts are likely adsorbed^44,45,64,65^. In contrast, in the present study, such adsorption is considered minimal because cabbages were cultivated inside a greenhouse and because only soft leaves inside a cabbage head were used for feeding.

This line of discussion leads to the second point. Wild-harvested plants were not used in the present study, but our experimental system employed the real Fukushima soil samples to cultivate cabbage heads from seeds. Thus, our experimental system would reflect the field conditions that the cabbage white butterfly had to cope with. Moreover, the quality of leaves was more controlled in the cultivated leaves than in the wild-harvested ones, excluding the possible regional genetic differences of the host plant leaves. Use of contaminated soils may not be advantageous to identify causal substances and mechanisms for biological effects because of the high heterogeneity and complex chemical composition of soils. To address this question, an addition of pure radioactive cesium to a defined cultivation medium may be a system of choice. However, use of the real contaminated soils is more relevant to understanding what has happened in the cabbage white butterfly and its associated ecosystems in Fukushima. In our experimental system, in addition to radiocesium, both unidentified substances from the Fukushima Dai-ichi nuclear power plant that could behave together with radiocesium and other anthropogenic or natural substances that could cause synergistic effects with radioactive substance would be included, which may be equally important as causal substances in the field.

Only small percentages of radiocesium were transferred to cabbage leaves from the soils used in this study, and the transfer rates varied considerably among the cabbage plants. This variability is not surprising and probably stemmed from various soil compositions^66^. Thus, we paired radioactivity concentrations of cabbage leaves (not of soils) for the correlation analyses with biological factors in the present study. Most importantly, our rearing system for the cabbage white butterfly can be viewed as a success because of its reasonably high adult achievement rate and total normality rate of the Okinawa group. Therefore, we are certain that our developmental and hemocytological data were not biased by an ill-defined rearing system or crude rearing practice. For correlation analyses, we excluded the Iitate samples due to their nonlinear behavior, but we believe that this nonlinear behavior was not an artifact and had nothing to do with the rearing system itself. In the future, not only radioactivity concentrations but also absorbed doses of larvae, prepupae, or pupae should be discussed.

Nonetheless, more larvae could not reach the prepupal, pupal, and adult stages in the contaminated groups than in the Okinawa group, as indicated by the adult achievement rate and the pupal eclosion rate. The developmental and hemocytological factors were measurable only in the individuals that survived at least up to the prepupal stage. In this sense, it is to be understood that the values for the developmental and hemocytological factors that were obtained here may be “underestimated” if these values are used to evaluate the biological effects of the Fukushima nuclear accident in the wild.

**Figure 4:**
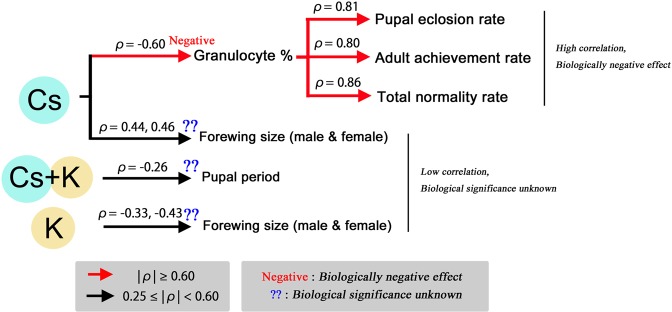
Summary and interpretation of correlation analysis. Radioactivity concentrations of cesium, potassium, and their summation are assumed to affect developmental and hematological factors. Similarly, changes in hemocyte percentages are assumed to affect developmental factors. Based on these assumptions and the results of the correlation analyses, directions of arrows indicate possible causal relationships.

Regarding the third point, the main findings of the correlation analyses are summarized in Fig. 4, assuming the causal contributions of radioactivity concentrations of cesium and potassium. The granulocyte percentage was negatively correlated with cesium radioactivity. This finding is important because it suggests a decrease in granulocytes in response to radiocesium concentration in the field. Because granulocytes are positively correlated with the pupal eclosion rate, adult achievement rate, and the total normality rate, an increase in the cesium radioactivity may cause a decrease of the number of granulocytes in hemolymph and then a decrease in the pupal eclosion rate, the adult achievement rate, and the total normality rate. Granulocytes are functional phagocytes in the cabbage white butterfly^59^ and might have engulfed radioactive debris. The lowered granulocyte percentage could mean that more granulocytes died in response to the increase in cesium radioactivity. Granulocytes might have undergone functional apoptosis when larvae ate contaminated leaves. Alternatively, differentiation into granulocytes may be inhibited by cesium radioactivity. Another possibility is that the decrease in granulocytes in response to cesium may be a consequence of the general stress response. Regardless, cesium effects may be direct on larval cells or indirect through the cabbage leaves. Changes in blood cells in response to low-levels of radiation exposures may have something in common between mammals^23,24,26^ and insects. In the field, less granulocytes in hemolymph would cause vulnerability of butterflies to invading substances, leading to a decrease in the number of butterflies and a change of their genetic composition at the population level. On the other hand, the plasmatocyte percentage had correlations only with the male and female forewing sizes, but their biological significance is not understood. The prohemocyte percentage was not correlated with any developmental or radioactivity factors.

In the previous experiments using the pale grass blue butterfly, the forewing size of irradiated individuals decreased^44^. In our field survey, a decrease in the forewing size in the pale grass blue butterfly was obtained only immediately after the accident (May 2011)^44^. The positive correlations of the cesium radioactivity with the forewing sizes of both males and females are not well understood in terms of biological significance, but we speculate that an acute high-level exposure may cause a decrease in the wing size, as shown in the silk worm moth^67^, whereas a chronic low-level exposure may cause an increase in the wing size.

Negative correlations of potassium radioactivity with the male and female forewing sizes were also detected, which was opposite from the cesium effects. We do not mechanistically understand these opposite effects of radiopotassium and radiocesium. Because the level of potassium radioactivity represents the entire amount of potassium species in nature, this negative correlation may simply indicate biochemical (nonradioactive) effect of potassium, considering that ^40^K comprises just 0.0117% of the entire potassium species^68^. However, if these results are not artifacts, this is directly related to the fourth point above and suggests that potassium and cesium behave differently in the butterfly despite their chemical similarity. Indeed, if potassium and cesium had behaved together, their radioactivity summation would have had more biologically relevant correlations in the present study. Although a weak ^40^K contribution as a summation of the cesium and potassium radioactivity to the pupal period was observed, its correlation coefficient was relatively low and not very convincing. This result could be an artifact because the pupal period was recorded only for individuals that eclosed; dead pupae during the pupal period were not included.

Indeed, the fourth point above is one of the most important in this study; the ^40^K radioactivity concentration was measured and analysed together with that of ^134^Cs + ^137^Cs. A linear relationship between radiocesium and radiopotassium radioactivity concentrations suggests that these elements behave similarly in being absorbed into cabbage, as expected from their chemical similarity. Despite their similarity, it is of great importance to stress that there was no significant correlation of ^40^K with developmental and hemocytological factors except the forewing size. This finding may not be surprising, considering that ^40^K occurs naturally. Nonetheless, this finding may be surprising, considering that the cabbage leaves used in the present study contained more ^40^K than ^134^Cs + ^137^Cs in terms of radioactivity concentration. It is probable that environmentally added anthropogenic radiocesium is more stressful for organisms than naturally occurring radiopotassium because naturally occurring radiopotassium would be tolerated through natural selection, but anthropogenic radiocesium would not be even at low levels. There is a body of evidence that Cs^+^ is much less permeable to potassium channels than K^+^ and acts as a blocker of potassium channels^69–74^. It can be speculated that radioactive Cs^+^ denatures potassium channels, whereas radioactive K^+^ does not. However, precise mechanisms of radiocesium effects are not clear. Since energy levels of emitted particles and their oral effective dose coefficients are very different between cesium and potassium^68,75^, a simple comparison in becquerel values may be invalid to differentiate biological effects of these radionuclides.

In the wild, radiation effects may be modified by other environmental stressors^76,77^. This synergistic effect may be widely found in nature; e.g., between nutrition and *Bt* toxins^78^. In the present study, the Ohara cabbage leaves contained lower radioactivity concentrations of cesium than the Baba cabbage leaves, but significant effects on development were detected only from Ohara. This finding, together with other results, may point out possibilities that the biological effects may not be explained solely by radioactivity concentrations and that biological effects of low-level radiation exposures may not be negligible for wild organisms. The mechanisms of the low-dose effects in the wild are not known, but one of the possibilities may be a biochemical change in plants induced by radiation stress that makes leaves toxic to insects^58^. Because butterfly larvae of most species are allowed to eat only the leaves on which larvae are deposited as eggs, larvae have no way of escaping these plant chemical effects. Consistent with this line of discussion, biological effects of Fukushima radiation on some plants have already been reported^28–31^. However, current knowledge of radiation biology is too immature to accurately predict any effects in the wild.

Because cabbage is a major vegetable eaten by humans, it is tempting to discuss what would occur if humans were to eat the contaminated cabbage used in this experiment, although cabbage with the contamination levels of this study would not be found in the market. We speculate that nothing more than a very minor stress response would occur in people who ingest cabbage with this level of contamination if our speculation on the modified plant chemical composition was correct. However, the possibility that a small number of people may be sensitive enough to become sick cannot be excluded, considering high sensitivity variation in humans and in other species^52,58,79^.

## Methods

### Butterflies and larval rearing

Throughout this paper, the cabbage white butterfly *P*. *rapae* (Linnaeus, 1758) was used. The Japanese subspecies of this butterfly species are known as *P*. *rapae crucivora* Boisduval, 1836^80^. Larvae of this butterfly eat cruciferous plants and prefer cultivated cabbage. All rearing procedures were performed in our laboratory, University of the Ryukyus, Okinawa, Japan. Throughout the rearing procedure, we controlled lighting under L18:D6 conditions at 25–27 °C.

We collected female adult individuals from Yomitan Village and Nanjo City, Okinawa-jima Island, the Ryukyu Archipelago, Japan. Eggs were collected in the laboratory in Okinawa from these field-collected females (*n* = 9). Egg-collecting was attempted 4 times (Table S6). We used an insect rearing cage (300 × 740 × 300 mm) (MegaView Science, Talchung, Taiwan), in which cabbage leaves (cultivated in Okinawa for control rearing) and female butterflies were placed together with some flowers for nectar such as Dahlberg daisy *Thymophylla tenuiloba*, sweet alyssum *Lobularia maritima*, and Spanish needle *Bidens pilosa* (Fig. 1d,e). Cabbage leaves inside the cage, on which eggs were deposited, were replaced with new ones daily until the female butterflies died (2–4 days). The egg period was approximately 3 days, after which larvae hatched. Larvae ate noncontaminated cabbage leaves on which they were born until the fifth day after egg collection. Then, the first-instar larvae were randomly allocated to one of the four groups to feed the contaminated or control cabbage leaves. Each group of larvae were fed the cabbage leaves of different contamination levels. Larvae from the same females were evenly allocated to all cabbage groups to avoid genetic bias among these groups. Therefore, any effects are attributable to cabbage quality and possibly the level of radioactive materials in the cabbage leaves, although nonradioactive materials could also contribute to the effects. Each group started with approximately 158 individuals for the rearing experiment and 28 individuals for hemolymph collection (Table S6). Throughout this study, only soft cabbage leaves inside the cabbage heads were used for egg collection and larval rearing procedures without washing.

The rearing experiment for the developmental factors and the forewing sizes were performed 3 times independently under the same protocol (trial numbers 1, 2, and 4). Developmental factors such as the total normality rate (see below) were calculated for each trial and were averaged using data from three trials. The forewing sizes were also averaged in this way, but the individual size data were also used for statistical analyses. The hemolymph collection was attempted twice (trial numbers 3 and 4) under the same protocol, and the percentages of hemocytes (see below) were averaged using these two trials.

For rearing, two types of plastic containers were used depending on larval size and density: a cylindrical container (55 mm height × 101 mm in diameter) and a square prism container (57 mm height × 168 mm width × 168 mm depth). Container cleaning and feeding processes were carried out every day or every two days, and simultaneously, dead bodies were identified, if any, and the number of individuals were counted. At the prepupal stage, each individual was independently transferred to a cylindrical plastic container (55 mm height × 101 mm in diameter) and was numbered individually. After eclosion inside the container, adults were readily frozen.

### Soil and cabbage cultivation

We cultivated the miniature cabbage “F_1_ Yokamaru” (Kokkaen, Osaka, Japan), a commercially available Japanese cultivar of the cabbage *Brassica oleracea* var. *capitate*, from seeds. Seeds for cultivation were used from a single commercial package, irrespective of the soil group, to minimize genetic variability as much as possible. We collected soils from Okinawa and Fukushima at the following localities: (1) Nanjo City, Okinawa Prefecture, as a control, (2) Ohara, Minami-soma City, Fukushima Prefecture, (3) Baba, Minami-soma City, Fukushima Prefecture, and (4) Iitate Village, Fukushima Prefecture. Four plastic planters (345 mm height × 520 mm width × 260 mm depth) per locality group were filled with the wild-collected soils mixed with a small amount of natural fertilizer.

During the early stages of cultivation, we used a kit including a nursery pot and soil (Jiffy 7) and a small greenhouse for seedlings (Moerdijk, Netherlands). We seeded 3 seeds per pot, and when 3 or 4 true leaves were formed, only one seedling was saved. When seedlings had 5 or 6 true leaves, the seedlings were planted in the planter containing the field-collected soils. Before the planting process, the field-collected soils were mixed with 100 g of fertilizer MagAmp K (HYPONeX, Osaka, Japan) per planter. Three and 6 weeks after the planting, 140 g of additional fertilizer My Garden (Sumitomo Chemical Garden Products, Tokyo, Japan) per planter was added. These seedling processes were performed in August and September with a one-month interval to accommodate 4 rearing attempts.

During the early stages of cultivation, planters were covered with fine-meshed nets to avoid insect damage. No insecticide was used throughout the cultivation period. Planters of the 3 soil groups from Fukushima Prefecture were placed in a plastic greenhouse in Minami-soma City, Fukushima Prefecture. Planters of the Okinawa soil group were placed in Nishihara Town, Okinawa Prefecture. The seeding cultivation was performed during the same period among 4 groups, and the Fukushima and Okinawa cabbage heads were harvested on the same day. The Fukushima cabbage was immediately sent to our laboratory in Okinawa under refrigeration. During the transportation period, the Okinawa cabbage was also stored in a refrigerator. During the feeding period, leaves were detached from the heads as necessary, and the remaining parts were stored in a refrigerator.

Our protocol eliminated the adsorption of radioactive materials on the surface of the cabbage leaves by two means. First, the planters were covered with fine-meshed nets during the early period of cultivation and were placed in a green house. Second, the outer leaves of the cabbage heads were not given to larvae. Only young leaves inside the cabbage heads were given to larvae.

### Developmental factors and morphological abnormalities

We obtained the following 5 developmental factors: the pupal eclosion rate (the number of individuals who eclosed among the number of pupae), the adult achievement rate (the number of individuals who successfully eclosed among the number of starting larvae used), the total normality rate (the number of adults without any noticeable morphological abnormality among the number of starting larvae), the larval period [days] (from the day of egg deposition to the pupation day including the prepupal stage; dead individuals during this period were not included), and the pupal period [days] (from the pupation day to the eclosion day; dead individuals during this period were not included, but individuals of eclosion failure were included) (Table S2). Morphological abnormalities were checked with the naked eye or using a conventional stereomicroscope. All individuals that successfully eclosed (individuals for which all body parts were escaped from the pupal case) were subjected to abnormality checks except for the individuals subjected to hemocyte counting. The adult abnormality rate (the number of abnormal adult individuals among the individuals that eclosed), which was reflected in the total normality rate, were also used when appropriate (Fig. 2d, Table S3). For the abnormality check, attention was paid to the following body parts: wing shape, appendages (legs, antennae, palpi, and proboscises), compound eyes, trunk (thorax and abdomen), and sexual organ at the tip of the abdomen (valva). Each type of abnormalities was given scores based on the frequency of that abnormality type across all samples, resulting in adult abnormality score for each group (see Statistical analyses; Fig. 2e, Table S3). It appeared that the color patterns were highly variable among individuals in this species despite exhibiting a small number of pattern elements. Variations in black spots and yellow areas in size and shape were considered within the normal range for this species. Abnormal structures were photographed using a high-resolution Keyence VHX-1000 digital microscope (Osaka, Japan).

### Forewing size measurements

The adult male and female forewing sizes were measured from the wing base to the apical point using a desktop digital microscope SKM-S30A-PC and its associated software SK measure (Saitoh Kougaku, Yokohama, Japan). The forewing was placed under a glass slide when necessary to make it flat. Both right and left forewing sizes were measured whenever possible, and the mean value was considered the final forewing size. In rare cases, only one forewing was intact. In that case, either right or left forewing size was considered the final forewing size.

### Hemocytological examinations

Larvae were reared for hemocytological examinations in the same way as above. Immediately after prepupation when the larva stopped moving, a dorsal side of an abdominal segment was cut to a depth of 1–2 mm. Bleeding hemolymph samples (4 μL from each individual) were collected. When the sample volume was less than 4 μL, an additional incision was made at a dorsal side of a different abdominal segment. The 4-μL hemolymph sample was readily mixed with 16 μL of Turk’s stain solution (Nakalai Tesque, Kyoto, Japan) to stain nuclei of hemocytes in light purple for cell counting. The mixture was then injected into two poring sites of a disposable OneCell® counter (OneCell, Nagahama, Shiga, Japan). After waiting one or two minutes for cell settlement, the cell counter was set under a high-resolution Keyence VHX-1000 digital microscope (Osaka, Japan), and images for all 8 counting compartments per sample were obtained. The number of hemocytes were then counted in these images using the cell counter function of ImageJ^81^. Cells having a contact with borderlines of compartments were not counted, according to the manufacturer’s protocol.

Hemocytes were classified into 3 cell types (granulocyte, plasmatocyte, and prohemocyte) based on their cellular morphology and staining patterns, according to Wago and Kitano (1985)^59^. Plasmatocytes are relatively large (8–15 μm in diameter) and flat cells with variable shapes that have round cytoplasmic inclusions, lamellipodia, and filopodia. Granulocytes are medium-sized (5–10 μm in diameter) and round or oval cells that have round cytoplasmic inclusions (more than plasmatocytes) and filopodia but no lamellipodia. Prohemocytes are relatively small (4–6 μm in diameter) and round or oval cells that have no cytoplasmic inclusions and no filopodia. Their percentages among all hemocytes were obtained, and these 3 factors were examined for possible correlations with radioactivity concentrations of radiocesium (^134^Cs + ^137^Cs), radiopotassium (^40^K), and the summation of both radiocesium and radiopotassium (^134^Cs + ^137^Cs + ^40^K). Hemocyte percentages were also paired with 5 developmental factors and male and female forewing sizes for correlation analyses.

The individuals used for hemolymph collection were not used for the rearing experiment to obtain developmental factors and forewing sizes (Table S6). The hemocytological data from the individual who ate cabbage leaves of a given dose were associated with the developmental and forewing size data from the same locality group. For this reason, for the correlation analyses of the hemocytological data including those for the forewing size correlations, the group-averaged values (and not the individual-associated values) were used after normalisation. The normalisation was performed by dividing the original data of a given trial by the Okinawa data of that trial. This normalisation supposedly eliminates strain-specific variability among samples. Since there were 3 rearing trials, 3 points from the Okinawa groups were all located at 1.00 in the scatter plots.

### Radioactivity measurements

A Canberra GCW-4023 germanium semiconductor device (Meriden, CT, USA) was used to measure radioactivity of the cultivated cabbage leaves. The cabbage leaves (either remnants that were fed larvae or leaves that were not fed larvae but from the same cabbage head as the fed leaves, excluding the hard leaf veins that would not be eaten by larvae) were completely air-dried via long-time confinement within a sealed container together with a desiccating agent.

Additional cabbage leaf samples were prepared to obtain the dry rate. To do this, samples were weighted before and after the drying procedures. The dry rate was 7.63%, which was used to calculate radioactivity concentrations in wet weight. Dried samples were grinded into small pieces and put into a columnar plastic vial before radioactivity measurements. Depending on the height of samples in a vial, the following counting efficiencies were employed to calculate radioactivity concentrations: For ^137^Cs, 19.9% (7–8 mm), 19.5% (10 mm), and 14.6% (35 mm); for ^40^K, 8.35% (7–8 mm), 7.93% (10 mm), and 5.73% (35 mm). From measured values on a particular measuring day, the radioactivity values on the very first day of feeding radioactive leaves were calculated, which were considered the radioactivity concentration of the fed cabbage. Branching ratios for ^137^Cs (662 keV) and ^40^K (1461 keV) used were 85.1% and 10.7%, respectively^68^. Radioactivity concentrations of ^134^Cs were calculated based on the ^137^Cs measurements, assuming that the ratio of ^137^Cs and ^134^Cs was 1:1 on March 15, 2011. In the case of the Okinawa samples, the ^134^Cs peak at 605 keV was not detected at all, and the ^137^Cs peak detected at very small levels were likely from nuclear fallout from nuclear bomb experiments in the 1950s. Thus, ^134^Cs and ^137^Cs were considered nonexistent in the Okinawa samples.

Radioactivity concentrations of ^137^Cs in soils from Okinawa used for cabbage cultivation were measured using the Canbarra GCW-4023 as above, and those from Fukushima were measured using a NaI (TI) scintillation detector FNF-401 (OHYO KOKEN KOGYO, Fussa, Tokyo, Japan). From measured values on a particular day, radioactivity concentrations of the day of the first planting of seedlings were calculated, and these were considered the radioactivity concentrations of soils.

### Statistical analyses

The statistical software R, version 3.2.1 (The R foundation for Statistical Computing, Vienna, Austria), was used to perform Pearson correlation analyses, Steel tests, and Spearman correlation analyses. For the Steel test shown in Fig. 2a, a value of 1 (successfully achieving the adult stage) or 0 (died before achieving the adult stage) was assigned to each individual. Similarly, for the Steel test shown in Fig. 2b, a value of 1 (successfully becoming a normal adult) or 0 (becoming an adult with morphological abnormality or died before achieving the adult stage) was assigned to each individual. For the Steel test shown in Fig. 2e, unique scores were assigned to each individual (Table S3), as calculated based on their frequencies after categorizing into 3 groups: the wing group, the head group (proboscis and palpus), and the body group (trunk and valva). In addition, the normal individuals were also assigned a unique score based on the same calculation. For example, the score for the wing abnormalities (12.0) was obtained as the total number of individuals examined (383) divided by the number of abnormalities (32). The scores for the Okinawa, Ohara, Baba, and Iitate groups were obtained by the summation of scores of all the individuals that belonged to those groups. When two different abnormalities were found in an individual, the summation of both scores was assigned to that individual.

For Spearman correlation analyses, individual-associated values were used for the forewing sizes when they were paired with radioactivity concentrations. However, the group-associated values were used to pair with the hemocytological factors because they were obtained from different individuals. For developmental and hemocytological factors, the original data of a given trial were divided by the Okinawa data of that trial for normalisation (indicated as “ratio” in Fig. 3). To avoid type I errors in performing the correlation analyses, Holm-adjusted *P*-values were obtained, those with *P* < 0.05 were considered significant, and these significant cases were reported with scatter plots in this study. However, the original non-corrected *P*-values were reported to understand inherent characteristics for their correlations.

## Supplementary information

Supplementary Information Tables S1–S6

## Data Availability

Almost all data generated or analysed in this study are included in this published article and its Supplementary Information files. The datasets not included in this article and its Supplementary Information files are available from the corresponding author on reasonable request.
