# Supplementary material for: Developmental and hemocytological effects of ingesting Fukushima’s radiocesium on the cabbage white butterfly Pieris rapae
Source: Sci Rep. 2019 Feb 22;9:2625. doi: 10.1038/s41598-018-37325-9 (PMC6385249; doi:10.1038/s41598-018-37325-9)
Supplement: Supplementary file 1 — Supplementary Information Tables S1–S6 [file 41598_2018_37325_MOESM1_ESM.pdf]

Supplementary Information

Developmental and hemocytological effects of ingesting Fukushima's radiocesium on the cabbage white butterfly *Pieris rapae*

Wataru Taira[1,2], Mariko Toki[1], Keisuke Kakinohana[1], Ko Sakauchi[1] & Joji M. Otaki[1]

[1] The BCPH Unit of Molecular Physiology, Department of Chemistry, Biology and Marine Science, Faculty of Science, University of the Ryukyus, Okinawa 903-0213, Japan
[2] Center for Research Advancement and Collaboration, University of the Ryukyus, Okinawa 903-0213, Japan

Correspondence and requests for materials should be addressed to J.M.O. (otaki@sci.u-ryukyu.ac.jp)

Tables S1-S6

Table S1. Radioactivity concentrations of cesium and potassium in cultivated cabbage leaves (wet).

Locality group	Trial number	Cesium (^{134}Cs + ^{137}C) [Bq/kg]		Potassium (^{40}K) [Bq/kg]	
		Measurements	Mean ± SD	Measurements	Mean ± SD
Okinawa (Nanjo City, Okinawa Prefecture)	1	ND	ND	213.7	139.2 ± 31.4
	2	ND		118.4	
	3	ND		108.7	
	4	ND		115.8	
Ohara (Minamisoma City, Fukushima Prefecture)	1	13.5	13.6 ± 2.1	95.9	96.6 ± 11.6
	2	11.1		108.4	
	3	13.9		101.2	
	4	16.1		81.0	
Baba (Minamisoma City, Fukushima Prefecture)	1	46.7	30.5 ± 15.7	117.4	118.9 ± 29.0
	2	38.7		156.1	
	3	10.9		85.2	
	4	25.6		117.0	
Iitate (Iitate Village, Fukushima Prefecture)	1	195.9	204.3 ± 74.8	184.9	178.7 ± 84.4
	2	311.2		293.0	
	3	140.1		95.8	
	4	170.1		141.2	

ND: not determined (below detection limit).

Table S2. Developmental factors examined in this study (pupal eclosion rate, adult achievement rate, total normality rate, larval period, and pupal period).

Locality group	Trial number	Pupal eclosion rate (%)	Adult achievement rate (%)	Total normality rate (%)	Larval period (day)	Pupal period (day)
Okinawa (Nanjo City, Okinawa Prefecture)	1	85.7	57.1	51.9	14.5±1.1	6.1±0.4
	2	100.0	86.2	86.2	14.2±1.1	6.0±0.2
	4	100.0	91.3	91.3	15.0±0.8	6.0±0.0
	Mean ± SD	95.2 ± 8.2	78.2 ± 18.4	76.5 ± 21.4	14.6±.1.4	6.1±0.1
Ohara (Minamisoma City, Fukushima Prefecture)	1	82.0	46.7	35.8	14.6±1.0	6.0±0.4
	2	75.0	53.6	50.0	14.4±0.8	6.0±0.0
	4	90.9	87.0	87.0	15.0±1.3	6.1±0.2
	Mean ± SD	82.6 ± 8.0	62.4 ± 21.5	57.6 ± 26.4	14.7±0.4	6.0±0.0
Baba (Minamisoma City, Fukushima Prefecture)	1	80.3	53.3	48.6	14.5±3.0	6.0±0.4
	2	100.0	85.7	85.7	14.3±1.0	6.0±0.0
	4	100.0	73.9	73.9	14.4±0.6	6.1±0.2
	Mean ± SD	93.4 ± 11.4	71.0 ± 21.5	69.4 ± 19.0	14.4±0.1	6.0±0.0
Iitate (Iitate Village, Fukushima Prefecture)	1	75.3	57.0	44.9	14.7±1.2	6.3±0.6
	2	96.3	89.7	82.8	14.4±1.3	6.0±0.0
	4	100.0	39.1	34.8	15.1±0.6	6.0±0.0
	Mean ± SD	90.5 ± 13.3	61.9 ± 25.6	54.1 ± 25.3	14.7±0.3	6.1±0.1

Table S3. Adult abnormality profiles and scores.

	Number of individuals					Abnormality score assigned
	Okinawa	Ohara	Baba	Iitate	Total	
Abnormalities in wings	5	9	4	14	32	12.0
Abnormalities in proboscises	0	2	1	1	4	76.6
Abnormalities in palpi	0	0	0	1	1	
Abnormalities in trunk	0	1	0	2	3	95.8
Abnormalities in valva	0	0	0	1	1	
Total abnormal regions	5	12	5	19	41	-
Abnormal individuals	5	12	5	16	38	-
Individuals with double abnormalities	0	0	0	3	3	-
Normal individuals	100	72	93	80	345	1.1
Adult samples examined	105	84	98	96	383	-
Abnormality rate [%]	4.8	14.3	5.1	19.8	10.7	-
Mean abnormality score per individual	1.6	5.2	2.3	7.3	4.0	-

Table S4. Adult wing size.

Locality group	Trial number	Male forewing size		Female forewing size	
		Number of individuals	Mean ± SD (mm)	Number of individuals	Mean ± SD (mm)
Okinawa (Nanjo City, Okinawa Prefecture)	1	27	24.6 ± 1.1	30	23.3 ± 1.1
	2	16	24.8 ± 0.9	9	24.2 ± 0.7
	4	12	24.2 ± 0.8	9	23.2 ± 0.5
	Mean ± SD	-	24.5 ± 0.3	-	23.5 ± 0.5
Ohara (Minamisoma City, Fukushima Prefecture)	1	26	26.1 ± 1.0	24	25.1 ± 1.2
	2	5	25.3 ± 0.9	10	25.4 ± 0.9
	4	8	25.2 ± 0.9	12	24.9 ± 0.5
	Mean ± SD	-	25.6 ± 0.5	-	25.2 ± 0.3
Baba (Minamisoma City, Fukushima Prefecture)	1	28	25.5 ± 1.1	29	24.8 ± 1.1
	2	11	26.1 ± 1.1	13	25.6 ± 0.8
	4	7	26.1 ± 0.6	10	25.3 ± 0.7
	Mean ± SD	-	25.9 ± 0.3	-	25.2 ± 0.4
Iitate (Iitate Village, Fukushima Prefecture)	1	37	25.8 ± 1.6	24	25.1 ± 1.6
	2	15	26.6 ± 1.0	11	26.1 ± 0.5
	4	5	25.9 ± 0.7	4	25.9 ± 0.4
	Mean ± SD	-	26.1 ± 0.5	-	25.7 ± 0.5

Table S5. Percentages of hemocyte types in hemolymph.

Soil group	Trial number	Number of Individuals used for counting	Hemocyte types		
			Plasmatocyte	Granulocytes	Prohemocytes
Nanjo (Okinawa Prefecture)	3	7	18.8±7.1	6.7±3.3	73.4±9.8
	4	6	9.3±5.2	11.1±8.5	79.6±5.1
Ohara (Minamisoma, Fukushima Prefecture)	3	11	15.1±11.1	4.2±1.9	80.7±10.1
	4	7	13.8±4.4	5.0±0.9	81.2±3.8
Baba (Minamisoma, Fukushima Prefecture)	3	8	29.3±15.2	7.0±4.1	63.7±14.5
	4	3	10.4±2.8	6.8±0.4	82.9±3.1
Iitate (Fukushima Prefecture)	3	4	23.4±11.5	5.5±5.5	71.2±16.9
	4	6	7.9±1.8	3.6±1.4	88.5±1.5

Table S6. Number of mother butterflies and collected eggs used for the rearing experiment and hemocyte counting experiment

Trial Number	Egg collection date (y/m/d)	N of mother butterflies		N of collected eggs	Used for analysis	
		Each day	Total	per locality group	Rearing	Hemocyte
1	2014/01/11-12	1	6	18 1)	✓	
	2014/01/12-13	6		50 2)	✓	
	2014/01/13-14	5		39 3)4)	✓	
2	2014/01/24-25	1	1	28 5)	✓	
3	2014/02/1-2	1	1	14		✓
4	2014/02/11-12	1	1	23	✓	
				12		✓

Deviations from the numbers listed above: 1) Okinawa 17, 2) Iitate 49, 3) Okinawa 38, 4) Iitate 40, 5) Okinawa and Iitate 29.
